# Early Alzheimer's disease diagnosis and blood biomarkers: primary care physician attitudes, perceptions and barriers in the USA

**DOI:** 10.1002/alz70856_097183

**Published:** 2025-12-24

**Authors:** Susan Alford, Sheena K. Aurora, Justine Coppinger, Martí Jiménez‐Mausbach, Rosemary D. Laird, Hemant Pandey, Sutapa Ray, Jeffrey M. Burns

**Affiliations:** ^1^ Novo Nordisk Inc., Plainsboro, NJ, USA; ^2^ C2N Diagnostics, LLC, Saint Louis, MO, USA; ^3^ Novo Nordisk A/S, Copenhagen, Capital region, Denmark; ^4^ Mymemoryclinic.org, Melbourne, FL, USA; ^5^ NAN Navigator Inc., Orlando, FL, USA; ^6^ Brain and Spine Center, Chandler, AZ, USA; ^7^ University of Kansas Alzheimer's Disease Research Center, Kansas City, KS, USA

## Abstract

**Background:**

Early and accurate diagnosis of Alzheimer's disease (AD) is critical for ensuring access to targeted lifestyle recommendations and disease modifying therapies (DMT), which can slow disease progression. Historically, diagnosis has been slow, complex, costly, inaccurate, and inconsistent, with individuals often diagnosed in later stages of the disease. Today, AD diagnosis is evolving from symptom‐based exclusion to biomarker‐based inclusion, reflecting the underlying biological etiology. Primary care physicians (PCPs) are expected to play an increasingly important role in AD diagnosis and care. Novel assays employing blood biomarkers (BBM) could support an early and accurate AD diagnosis and be more accessible in primary care than traditional diagnostic tools, including positron‐emission tomography (PET) or cerebrospinal fluid (CSF) biomarker analysis. This study aimed to uncover PCPs’ attitudes on, perceptions of and barriers to AD diagnosis and incorporating BBMs into the diagnostic workflow.

**Method:**

Remote 60‐minute in‐depth interviews with 20 PCPs were conducted May 12‐26, 2023. Participants included generalists and geriatricians representing urban, suburban and rural US practices. Interviews focused on early AD diagnosis, PCP role and referral, BBMs, and key barriers or requirements for their clinical implementation.

**Result:**

Most PCPs believe that investigating cognitive decline is an important part of their role and are somewhat confident in diagnosing AD but would like to improve their skills. PCPs also report facing barriers such as the complexity and time‐consuming nature of current diagnostic methods, lack of effective treatments and the stigma of an AD diagnosis. PCPs responded positively to BBMs, viewing them as accurate and cost‐effective tools that could integrate easily into their practice. However, they expressed concerns about reimbursement for BBMs and the need for clarity on their place in the diagnostic pathway.

**Conclusion:**

This study highlights PCPs’ engagement and interest in supporting AD diagnosis as well as their receptivity towards BBMs. PCPs are generally positive about integrating BBMs into their clinical practice. However, clarity on healthcare coverage and context of use is needed before adoption. Continued medical education on diagnosing AD as well as interpretation and communication of BBM test results will be beneficial for primary care involvement in AD diagnosis.

